# The neuropsychopharmacology of acetyl-L-carnitine (LAC): basic, translational and therapeutic implications

**DOI:** 10.1007/s44192-023-00056-z

**Published:** 2024-01-02

**Authors:** Benedetta Bigio, Shofiul Azam, Aleksander A. Mathé, Carla Nasca

**Affiliations:** 1 Department of Psychiatry, New York University Grossman School of Medicine, New York, NY USA; 2 Nathan S. Kline Institute for Psychiatric Research, Orangeburg, NY USA; 3 Department of Clinical Neuroscience, Karolinska Institutet, Stockholm, Sweden; 4 Department of Neuroscience and Physiology, New York University Grossman School of Medicine, New York, NY USA; 5 Neuroscience Institute, New York University Grossman School of Medicine, New York, NY USA

**Keywords:** Histone acetylation, Glutamate, Hippocampus, Depression, Cognition

## Abstract

Mitochondrial metabolism can contribute to nuclear histone acetylation among other epigenetic mechanisms. A central aspect of this signaling pathway is acetyl-L-carnitine (LAC), a pivotal mitochondrial metabolite best known for its role in fatty acid oxidation. Work from our and other groups suggested LAC as a novel epigenetic modulator of brain plasticity and a therapeutic target for clinical phenotypes of depression linked to childhood trauma. Aberrant mitochondrial metabolism of LAC has also been implicated in the pathophysiology of Alzheimer’s disease. Furthermore, mitochondrial dysfunction is linked to other processes implicated in the pathophysiology of both major depressive disorders and Alzheimer’s disease, such as oxidative stress, inflammation, and insulin resistance. In addition to the rapid epigenetic modulation of glutamatergic function, preclinical studies showed that boosting mitochondrial metabolism of LAC protects against oxidative stress, rapidly ameliorates insulin resistance, and reduces neuroinflammation by decreasing proinflammatory pathways such as NFkB in hippocampal and cortical neurons. These basic and translational neuroscience findings point to this mitochondrial signaling pathway as a potential target to identify novel mechanisms of brain plasticity and potential unique targets for therapeutic intervention targeted to specific clinical phenotypes.

This article describes research in our and other laboratories on mitochondrial metabolism of acetyl-L-carnitine (LAC) that has led to the discovery of novel epigenetic mechanisms for the rapid regulation of brain plasticity in multiple rodent models and then has prompted us to uncover a role for this proposed mitochondrial signaling pathway of epigenetic function as a therapeutic target for clinical phenotypes of depression linked to childhood trauma, and implications for Alzheimer’s disease (Fig. 1). Multiple preclinical and clinical studies showed that epigenetic mechanisms are involved in the pathophysiology and treatment of stress-related depressive and cognitive disorders; the reversible properties of epigenetic modifications posit them as emerging potential targets for next-generation therapeutic interventions [1–5]. The goal is to recognize those biological changes that underlie aberrant epigenetic programming of brain plasticity, and to recognize mitochondrial signaling pathways, metabolic factors, transcriptomic profiles and structural changes that indicate flexible adaptability or the lack thereof. A key concept for understanding this interface is the model of allostasis (adaptation) and allostatic load (pathophysiology) [6] that we review below examining this model in relation to new insights from the recent work on the link between mitochondrial metabolism and epigenetic function to promote healthy behaviors and cognitive function.

**Fig. 1:**
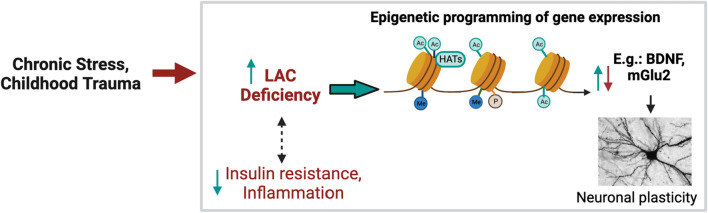
Mitochondrial health and epigenetic regulation of brain plasticity. Growing literature showed that mitochondrial metabolism can regulate histone modifications among other epigenetic mechanisms. A central tenant of this signaling pathway is acetyl-L-carnitine (LAC), a novel epigenetic modulator of brain plasticity and a therapeutic target for clinical phenotypes of depression linked to childhood trauma

## Learning from the past to promote present and future healthy trajectories

Over the past decades, a wealth of findings rekindled the interest in the older concept of Epigenetics (“Above the genome”), which was coined by Conrad Waddington to explain how identical genotypes could unfold different phenotypes as development proceeds [7, 8]. Since its inception in the early 1940s, Waddington’s concept of epigenetics took on additional meanings until encompassing the current notion of stochastic or environment-induced modifications of histone proteins or DNA without changes in the DNA sequence itself that alter the accessibility of genes to transcription factors [9]. In contrast to genetic mutations, epigenetic modifications rapidly affect target cell types exerting a long-lasting control in gene expression and provide a mechanism for a “non-genomic” inheritance. Epidemiological studies showed that genes contribute to the vulnerability of multiple CNS disorders, such as major depressive disorders (MDD), schizophrenia and substance use disorders with heritability respectively of 31–42%, 64–80%, and 23–79% [10–13]. Monozygotic twin studies, which allow an assessment of the epigenome (gene x environment) independent of underlying genomic sequence mutations, showed high discordance rates and related epigenetic changes [14] in monozygotic twins discordant for psychiatric illnesses. In addition to the high discordance rates between monozygotic twins, the importance of epigenetic mechanisms is evident in the chronic relapsing nature of these diseases, and the higher incidence of MDD in women after puberty [15, 16]. Genome wide association studies identified an increasing number of common variants associated with these diseases; however these variants explained only a small percentage of the genetic variation of the disorders, further supporting the concept that epigenetic processes play an important role [17].

## Epigenetic allostasis model

As an inescapable factor of the natural world, stress plays a pivotal role in the adaptive and maladaptive responses to the environment through epigenetic mechanisms [1, 18]. The brain is constantly being shaped, wittingly and unwittingly, by environmental forces and determines what is threatening to orchestrate behavioral and other physiological responses to life experiences [19]. Central to this concept linking neural and systemic functions is the concept of allostasis (adaptation) and allostatic load/overload (pathophysiology) [6] that emphasizes the role of endogenous mediators for adaptation in contributing to pathologies, including MDD, when activated persistently or dysregulated under circumstances of toxic stress and health-damaging behaviors (poor diet, excessive alcohol consumption, sleep deprivation and circadian disruption). In recent years, we added an epigenetic component to the Allostasis/Allostatic load model to describe how non-shared environmental experiences may set each individual on a somewhat different trajectory of development that determines either adaptive or maladaptive responses (behavior, systemic function and vulnerability to disease) to subsequent novel experiences as the life course unfolds [20]. We referred to this model as the Epigenetic Allostasis model.

We showed that inherent anxiety at baseline and elevated expression of the mineralocorticoid receptors (MR) in the hippocampus predisposes to a stress-induced suppression of expression of metabotropic glutamate receptors-2 (mGlu2, a key inhibitor of spontaneous glutamate release) with development of depressive-like behavior via epigenetic mechanisms of histone acetylation [20]. The nature of the experiences of the animals that develop increased MR expression with the related regulation of mGlu2 receptors is not known but might involve epigenetic experiences early in life, such as maternal care and stressors in the neonatal nesting environment. Individual differences in anxiety-like behaviors among genetically similar Sprague Dawley and Lewis male rats, living in the same environments and not previously exposed to experimental manipulations, have also been shown to predict lifespan and prefrontal cortical dendritic length [21, 22]. Furthermore, preclinical studies showed that juvenile stress increases hippocampal MR mRNA levels and anxiety-like behavior in adulthood [23]. Administration of spironolactone, a selective MR antagonist, counteracted stress-induced mGlu2 suppression and the related development of depressive-like behavior, implicating rapid action of glucocorticoids via a receptor that is known to modulate glutamate release [20]. An overflow of glutamate (the main endogenous excitatory amino in brain, at concentration of 5–15 mmol glutamate per kg body weight, higher than of any other neurotransmitter) is implicated in major psychiatric illnesses [1, 24–26]. Blocking MR receptors and interfering with glucocorticoids stimulation of glutamate activity counteracted stress-induced suppression of the histone acetyltransferase p300 mRNA levels in the hippocampus, showing a link between glucocorticoids and epigenetic programming of the glutamatergic system [20]. These growing preclinical studies of individual differences in stress response among inbred rodents further support the link between epigenetic mechanisms and development of individual phenotypes [20, 21, 27].

## Mitochondrial modulation of epigenetic function

There is an increasing recognition that mitochondrial metabolism can significantly affect epigenetic function and the corresponding brain plasticity. A key tenant of this emerging signaling pathway is LAC, an endogenous molecule that acts as a donor of acetyl groups to histone proteins and facilitates the transfer of fatty acids from cytosol to mitochondria during β-oxidation [28, 29]. Endogenous LAC levels range between 8 and 9 μg/g wet rodent tissue corresponding to 40–45 nmol/g wet tissue as previously reported in the rodent brain [30, 31]. We refer to a prior review for the pharmacology of LAC [28]. Prior studies showed that LAC levels increase rapidly after 500 mg i.v. administration to healthy volunteers [32]; oral administration of LAC leads to a significant increase in LAC concentrations in both plasma and cerebrospinal fluid (CSF) [33], showing that LAC crosses the blood–brain barrier and reaches the brain at significant concentrations.

In multiple rodent models of chronic stress (a shared risk factor for major psychiatric illnesses), administration of LAC leads to a rapid and persistent antidepressant-like response by increasing histone acetylation and expression of key genes, including mGlu2 receptors and the downstream brain-derived neurotrophic factor BDNF [27, 30, 34–37]. In cell culture systems, LAC formed in mitochondria is transported into cytosol and enters nucleus becoming a source of acetyl groups for histone acetylation [38]. Dysfunction of glutamatergic neurotransmission is a core feature of stress-related disorders, including MDD [27–29]. Administration of MS-275, an HDAC inhibitor, also increases expression of mGlu2 receptors, supporting the role of epigenetic mechanisms in the regulation of mGlu2 receptors [30]. The key role of histone acetylation in mGlu2 receptor regulation is also strengthened by the findings that chronic treatment with atypical antipsychotics down-regulates the expression of mGlu2 receptors in the mouse and human prefrontal cortex by increasing the binding of HDAC2 to the Grm2 promoter [39]. With regard to the relationship between BDNF and depressive disorders, there is evidence that expression and activity of BDNF in the hippocampus are decreased in response to stress and increased by antidepressant treatment [40–43]. Furthermore, postmortem studies showed increased BDNF expression in hippocampus after antidepressant treatment [44]. Thus, the action of LAC on BDNF levels and the rapid regulation of the glutamatergic system is in line with its antidepressant activity.

The antidepressant response to LAC occurs after 3 days of administration and lasts for at least 14 days, whereas the action of standard antidepressant drugs (fluoxetine and clomipramine) requires 14 days of consecutive administration and disappears after drug withdrawal in the same rodent models [30, 34]. We and others showed that administration of LAC leads to a rapid regulation of plasticity of brain areas such as the hippocampus and the connected prefrontal cortex, which are implicated in MDD and are among the first brain structures to degenerate in Alzheimer’s Disease (AD) [26, 30, 34–36, 45]. In addition to the rapid and long-lasting antidepressant response, boosting mitochondrial metabolism of LAC leads to the amelioration of specific cognitive domains [46, 47], and promotes behavioral resilience at the social defeat stress (SDS) paradigm [27]. The findings of the rapid and long-lasting antidepressant effects of LAC (which increases the expression of mGlu2, inhibitor of spontaneous glutamate release) supports the idea that targeting the glutamate system can lead to rapid antidepressant effects in agreement with the prior discovery of the antidepressant action of ketamine [48–50]. These findings compel further research to understand how mitochondrial metabolism and the related epigenetic programming of brain plasticity can serve as new targets to develop mechanism-based treatment models for psychiatric and neurodegenerative diseases.

## From basic neuroscience discoveries to translational innovation

In subjects suffering from MDD, plasma levels of LAC are decreased as compared to age- and sex-matched controls; the degree of LAC deficiency reflected both the severity and age of onset of MDD [51–54]. The lowest levels of LAC in severe clinical phenotypes of treatment resistant depression were also associated with emotional trauma in childhood, but none of the other subscales of the childhood trauma [52]. The greater decrease in LAC levels in severe forms of MDD and in patients with treatment resistant depression (TRD) is akin to a “kindling-like” progression of depression in that earlier age at onset and/or the presence of early life adversity, such as emotional trauma, conveys liability to more severe and treatment-resistant course of illness [51, 53, 55]. The specificity of these effects is in agreement with prior studies showing that the consequences of emotional maltreatments in childhood differ from those of physical and sexual abuse [56, 57]. Those subjects with a deficiency of LAC were previously characterized by an inflammatory tone as assessed by peripheral cytokine IL-6 levels [58]. In the initial study showing a link between LAC levels and clinical phenotypes of depression, patients were in an acute depressive episode at the time of study participation and the presence of medications did not influence LAC levels [51]. Future studies might help in elucidating trait-dependent LAC levels. Taken together with the findings that utilization of esketamine as an antidepressant increases LAC levels [59], the fact that the decrease in LAC levels in subjects with MDD is independent of psychotropic drug treatment raises the possibility that increasing LAC levels may be needed to induce antidepressant effects.

In our translational work, we also reported a link between LAC levels, cellular aging and metabolic function in the antidepressant responses to the insulin-sensitizing agent pioglitazone, an agent for treatment of diabetes type 2 and tested as potential antidepressant [60]. The decreased levels of LAC in subjects with MDD are linked to shorter leukocyte telomere length (LTL), increased body mass index (BMI), and higher reported rates of childhood trauma and are predictive of lack of antidepressant responses as assessed using the Hamilton depression rating scale-21. Conversely, those subjects with increased LAC levels, longer LTL, decreased BMI, and lower reported rates of childhood trauma showed an improvement in depressive symptoms in response to pioglitazone. These multidimensional factors spanning mitochondrial metabolism, cellular aging, metabolic function, and childhood trauma provided more detailed signatures to predict longitudinal changes in depression severity than each individual factor alone. We postulate that boosting mitochondrial metabolism might be a potential therapeutic strategy to induce antidepressant effects. In a double-blind, randomized, controlled clinical trial, LAC was not inferior to amisulpride in relieving symptoms of dysthymic patients, showing an excellent profile of safety and tolerability [61]. LAC has also shown efficacy in a limited cohort of senile patients with depression [62]. It is also important to note that LAC levels are decreased in obese subjects [63], emphasizing the need for future studies aimed at exploring a possible link between LAC metabolism and obesity in subjects with MDD. Future research is also needed to explore the antidepressant effects of LAC acting as part of a system network with other important mediators of brain plasticity in comparison with classical antidepressant drugs with particular focus on the effects in specific phenotypes of depression well-characterized not only for the individual endogenous levels of LAC but also for the corresponding changes in metabolic and inflammatory pathways as well as for history of adverse childhood experiences.

In the connection between mitochondrial metabolism and aging, prior work reported decreased levels of LAC in subjects with Alzheimer’s disease (AD) as compared to cognitively healthy controls, with intermediate levels in subjects with subjective memory complaint or mild cognitive impairment [64]. It is also important to note that a prior randomized, placebo-controlled, double-blind study of LAC treatment for probable AD patients showed that administration of LAC was associated with lower AD-related deterioration as assessed using the clinical dementia rating scale and mini-mental scale examination in those subjects younger than 65 years [65, 66]. While prevention is paramount, this growing mechanistic framework raises hypotheses for future studies that targeting mitochondrial metabolism can open windows of epigenetic plasticity toward more positive health outcomes, particularly in subjects with a history of adverse childhood experiences.

## Mitochondrial metabolism, inflammatory tone and insulin resistance

As mentioned above, LAC is an endogenous compound essential for β-oxidation and is an emerging epigenetic modulator of glutamatergic function. In addition to a decrease in peripheral levels of LAC, LAC levels are decreased by 40–60% in the hippocampus and prefrontal cortex of rodents with depressive-like behaviors, including the Flinders Sensitive Line (FSL) rats [30]. Work from our and other groups showed that LAC also modulates acetylation of NF-ĸB/p65 subunit, which potentiates the transcriptional activity of NF-κB member to increase transcription of the Grm2 gene encoding for the mGlu2 receptor in hippocampus and prefrontal cortex of FSL rats [30, 36]. The mGlu2 receptor promoter harbors numerous NF-κB responsive elements, as opposed to the promoter of the cognate receptors, mGlu3. In the FSL rats, a combined treatment with sodium salicylate, which is a non-selective inhibitor of NF-κB, counteracted the antidepressant effect of LAC on the expression of mGlu2 receptors, supporting the link between LAC-mediated mGlu2 induction and p65/NF-κB [30]. In the connection to inflammation, we showed that elevation of pro-inflammatory cytokine IL-6, increased anxiety at the light–dark test, and smaller hippocampal volume characterized at baseline (before any applied stress) those mice that become susceptible after social defeat stress (SDS) with social withdrawal and impaired transcriptomic-wide changes in ventral dentate gyrus, and that administration of LAC promoted behavioral resilience at the SDS paradigm in this susceptible phenotype [27]. Translational research further corroborates the link between a deficiency of LAC in clinical phenotypes of TRD and certain aspects of inflammation [51, 58].

In the connection to metabolism, boosting mitochondrial metabolism of LAC rapidly regulates central and peripheral insulin resistance (IR) [67], which is metabolic dysfunction implicated in the pathophysiology of both MDD and AD [68]. Using ^11^C PET, prior studies reported relatively high uptake of ^11^C labeled LAC-related acetyl groups; and this uptake was suppressed by i.v. administration of glucose, suggesting a link between LAC-related transport of acetyl groups and glucose signaling pathways, especially under conditions of metabolic stress [69]. Translational studies using exosomes showed a relationship between the epigenetic modulation of glutamatergic function and a brain metabolic dysfunction known as insulin resistance (IR) as showed by an increase and sex-specific phosphorylation in the expression of IRS1, a key marker of the insulin signaling cascade, in discrete exosomes enriched for the neural cell adhesion molecule L1 (L1CAM), a protein highly expressed in the brain [52, 70–72]. Our results showed a specificity of changes in L1CAM exosomes but not in total circulating exosomes in subjects with MDD. Hierarchical clustering analyses showed an association between brain IR and specific clinical symptoms, with the highest levels of IRS-1 in L1CAM exosomes associated with suicidality, anhedonia, depressed mood and feelings of guilt. A growing literature suggests that IR—which is ameliorated by boosting mitochondrial metabolism of LAC in rodent models—is one of the proposed steps in the irreversible activation of the cascade leading from mood disorders to AD [73–76]. These translational findings are an outgrowth of a mechanistic model in rodents with impaired plasticity of key brain areas relevant to mood, cognitive and other main disorders, wherein LAC levels are markedly decreased and signal abnormal brain and systemic functions.

These findings provided the closest available in vivo molecular signature for brain IR in depression and showed important sex differences in these pathways. The sex difference in serine-312 phosphorylation of IRS-1 (pSer-IRS-1) may be akin of an increased vulnerability and a more advanced stage of brain IR in women than men with MDD. Consistent with this postulate, we observed the greater increase in pSer-IRS-1 in women with stronger severity of depressive symptoms independently of use of psychotropic medications. Of note, the higher vulnerability of women than men to develop mood disorders begins about at the onset of puberty with the beginning of reproductive life [15, 16]. This sex difference disappears after menopause with the incidence of mood disorders becoming similar in men and women, supporting a key role of estrogen in mood disorders [77]. Prior studies showed that 17β-estradiol protects neurons from developing IR, and that ovarian hormones also contribute to regulate phosphorylation of IRS-1 [78]. Thus, multiple biological mechanisms may be involved in the sex difference in pSer-IRS-1 in L1CAM exosomes isolated from subjects with MDD. Clinical and epidemiological studies support sex difference in markers of IR in MDD showing that the prevalence of MDD is twice as higher in women than in men. There are also important sex differences in multiple IR-related chronic syndromes, such as obesity, diabetes, and cardiovascular disease that should be further explored in connection to LAC levels.

## Windows of epigenetic plasticity to re-direct health trajectories to positive outcomes

In summary, there appears to be a common denominator in the trajectories of stress-related disorders that we propose involves an epigenetic embedding of early life experiences through the mitochondrial metabolite LAC acting as part of a critical network system with other important mediators of brain plasticity and function, and that, when supplemented, rapidly alters gene expression profiles to ameliorate behaviors and cognitive function in animal models deficient in LAC because of stress-induced causes. While it is not possible to “roll back the clock”, deeper understanding of the biological pathways and mechanisms through which adverse childhood experiences produce a lifelong vulnerability to altered mitochondrial metabolism and the related pathways can provide a path for compensatory plasticity toward more positive health directions. Of note, a growing number of studies support mitochondrial metabolism of LAC as a common culprit underlying psychiatric and neurodegenerative diseases such as MDD and AD as well as obesity, making it important to further understand mechanisms for the development of aberrant mitochondrial metabolism of LAC. A key concept for understanding this interface is that while health-damaging behaviors (e.g.: poor diet, excessive alcohol consumption, sleep deprivation and circadian disruption) contribute to allostatic load and the many consequences of such behaviors on triggering and exacerbating these illnesses, it is increasingly recognized that health-promoting behaviors that protect mitochondrial metabolism and energy regulation are an essential component of successful allostasis.
